# Clinical Characteristics and Outcomes of Patients Hospitalized With COVID-19 During the First 4 Waves in Zambia

**DOI:** 10.1001/jamanetworkopen.2022.46152

**Published:** 2022-12-13

**Authors:** Peter A. Minchella, Duncan Chanda, Jonas Z. Hines, Sombo Fwoloshi, Megumi Itoh, Davies Kampamba, Robert Chirwa, Suilanji Sivile, Khozya D. Zyambo, Simon Agolory, Lloyd B. Mulenga

**Affiliations:** 1 Centers for Disease Control and Prevention–Zambia, Lusaka, Zambia; 2 Zambian Ministry of Health, Lusaka, Zambia

## Abstract

**Importance:**

Few epidemiologic studies related to COVID-19 have emerged from countries in Africa, where demographic characteristics, epidemiology, and health system capacity differ from other parts of the world.

**Objectives:**

To describe the characteristics and outcomes of patients admitted to COVID-19 treatment centers, assess risk factors for in-hospital death, and explore how treatment center admissions were affected by COVID-19 waves in Zambia.

**Design, Setting, and Participants:**

This retrospective cohort study assessed patients admitted to COVID-19 treatment centers in 5 Zambian cities between March 1, 2020, and February 28, 2022.

**Exposures:**

Risk factors for in-hospital mortality, including patient age and severity of COVID-19, at treatment center admission.

**Main Outcomes and Measures:**

Patient information was collected, including inpatient disposition (discharged or died). Differences across and within COVID-19 waves were assessed. Mixed-effects logistic regression models were used to assess associations between risk factors and in-hospital mortality as well as between characteristics of admitted patients and timing of admission.

**Results:**

A total of 3876 patients were admitted during 4 COVID-19 waves (mean [SD] age, 50.6 [19.5] years; 2103 male [54.3%]). Compared with the first 3 waves (pooled), the proportion of patients who were 60 years or older admitted during wave 4, when the Omicron variant was circulating, was significantly lower (250 of 1009 [24.8%] vs 1116 of 2837 [39.3%]; *P* < .001). Factors associated with in-hospital mortality included older age (≥60 vs <30 years; adjusted odds ratio [aOR], 3.55; 95% CI, 2.34-5.52) and HIV infection (aOR, 1.39; 95% CI, 1.07-1.79). Within waves, patients who were admitted during weeks 5 to 9 had significantly higher odds of being 60 years or older (aOR, 2.09; 95% CI, 1.79-2.45) or having severe COVID-19 at admission (aOR, 2.49; 95% CI, 2.14-2.90) than those admitted during the first 4 weeks.

**Conclusions and Relevance:**

The characteristics of admitted patients during the Omicron wave and risk factors for in-hospital mortality in Zambia reflect data reported elsewhere. Within-wave analyses revealed a pattern in which it appeared that admission of higher-risk patients was prioritized during periods when there were surges in demand for health services during COVID-19 waves. These findings support the need to expand health system capacity and improve health system resiliency in Zambia and other countries with resource-limited health systems.

## Key Points

**Question:**

What were the characteristics and outcomes of patients admitted to COVID-19 treatment centers during Zambia’s first 4 COVID-19 waves?

**Findings:**

In this cohort study of 3876 patients, across-wave analyses of data, including identification of risk factors for in-hospital mortality in Zambia, were in line with data reported elsewhere. Within-wave analyses revealed a pattern in which it appeared that admission of higher-risk patients was prioritized during periods of surging demand for health services during COVID-19 waves.

**Meaning:**

These findings suggest that increased efforts to expand health system capacity and improve health system resiliency in Zambia and other countries with resource-limited health systems are warranted.

## Teaser

This cohort study examines patient characteristics and risk factors for in-hospital death among patients admitted to COVID-19 treatment centers in Zambia during the COVID-19 epidemic.

## Introduction

Like many countries, Zambia is experiencing a COVID-19 epidemic characterized by waves in which steep increases in SARS-CoV-2–positive cases are closely followed by surges in patients with COVID-19 requiring hospitalization. The timing and public health impacts of waves are influenced by a variety of factors, including natural and vaccine-induced immunity,^1^ waning of such immunity,^2^ seasonality,^3^ mitigation measures,^4^ large events,^5^ and SARS-CoV-2 variant characteristics.^6,7,8,9^

Many of the differences between waves can be attributed to the ability of SARS-CoV-2 to mutate and produce characteristics that favor increased transmissibility and immune evasion.^10,11^ Some variant strains have also demonstrated increased (or decreased) virulence.^6,7,8,9^ When a new variant emerges or a new wave begins, collection and analysis of epidemiologic data provide useful information for researchers interested in understanding how new mutations affect disease pathogenesis and for public health officials assessing how the variant will affect (or has affected) health systems. Reports describing such analyses are available,^9,12,13,14,15^ but few are from countries in sub-Saharan Africa, where demographic characteristics, epidemiology, and health system capacity differ from other parts of the world.

Understanding how epidemiologic data collected in Zambia changed across and during COVID-19 waves also has potential to help inform future public health responses. The influence of health system capacity is particularly important to elucidate, because limited health system capacity directly affects downstream epidemiologic indicators during periods of surging demand (eg, hospitalization rates may be affected when hospitals are full, and case fatality proportions may be deflated when community COVID-19 deaths occur). In this report, we examine the characteristics of patients admitted to COVID-19 treatment centers during the COVID-19 epidemic in Zambia, assess risk factors for in-hospital death, and explore how characteristics of admitted patients were affected by periods of surging demand.

## Methods

### COVID-19 Clinical Outcomes Study

We conducted a retrospective cohort study of patients admitted to COVID-19 treatment centers in Zambia from March 1, 2020, through February 28, 2022. Beginning in March 2020, patients diagnosed with probable or confirmed COVID-19 in Zambia were admitted to Ministry of Health COVID-19 treatment centers that had specifically designated isolation and treatment units staffed by clinicians and nurses trained in COVID-19 clinical management. The study was conceived during the early weeks of the COVID-19 epidemic in Zambia and eventually included COVID-19 treatment centers in 5 of Zambia’s largest cities: Lusaka (4 treatment centers), Ndola, Kitwe, Kabwe, and Livingstone.^16^ The study protocol was approved by the University of Zambia Biomedical Research Ethics Committee. The study protocol was determined to be research according to the Centers for Disease Control and Prevention (CDC) human research protection procedures, but the CDC investigators did not interact with the study participants or have access to identifiable data or specimens for research purposes. Patients provided verbal consent for care and inclusion in the study. This study followed the Strengthening the Reporting of Observational Studies in Epidemiology (STROBE) reporting guideline.

Per national guidelines, admission to COVID-19 treatment centers was recommended for all patients with severe respiratory illness or those with both mild respiratory illness and risk factors for severe disease.^17^ Patients at participating treatment centers had demographic and clinical data collected at admission and during hospitalization until they were discharged or died. Data were collected using a standardized case record form adapted from the World Health Organization^18^ and entered into REDCap (Research Electronic Data Capture) electronic data capture tools by trained staff at a later date.^19,20^ Severe COVID-19 was defined as having an oxygen saturation level below 90%, having a respiratory rate greater than 30 breaths/min, or needing oxygen therapy.^21^ HIV status was self-reported, and patients with unknown status or who were deemed eligible and consented for HIV testing were tested at admission. Comorbidities were self-reported and included cardiac disease, hypertension, diabetes, other pulmonary disease, active tuberculosis, previous tuberculosis, asthma, kidney disease, liver disease, neurologic disorder, asplenia, malignant neoplasm, and current smoking. The number of comorbidities (not including HIV infection) was summed for each patient.

### National Testing Data, Wave Definitions, and Circulating Variants

**Figure 1.**
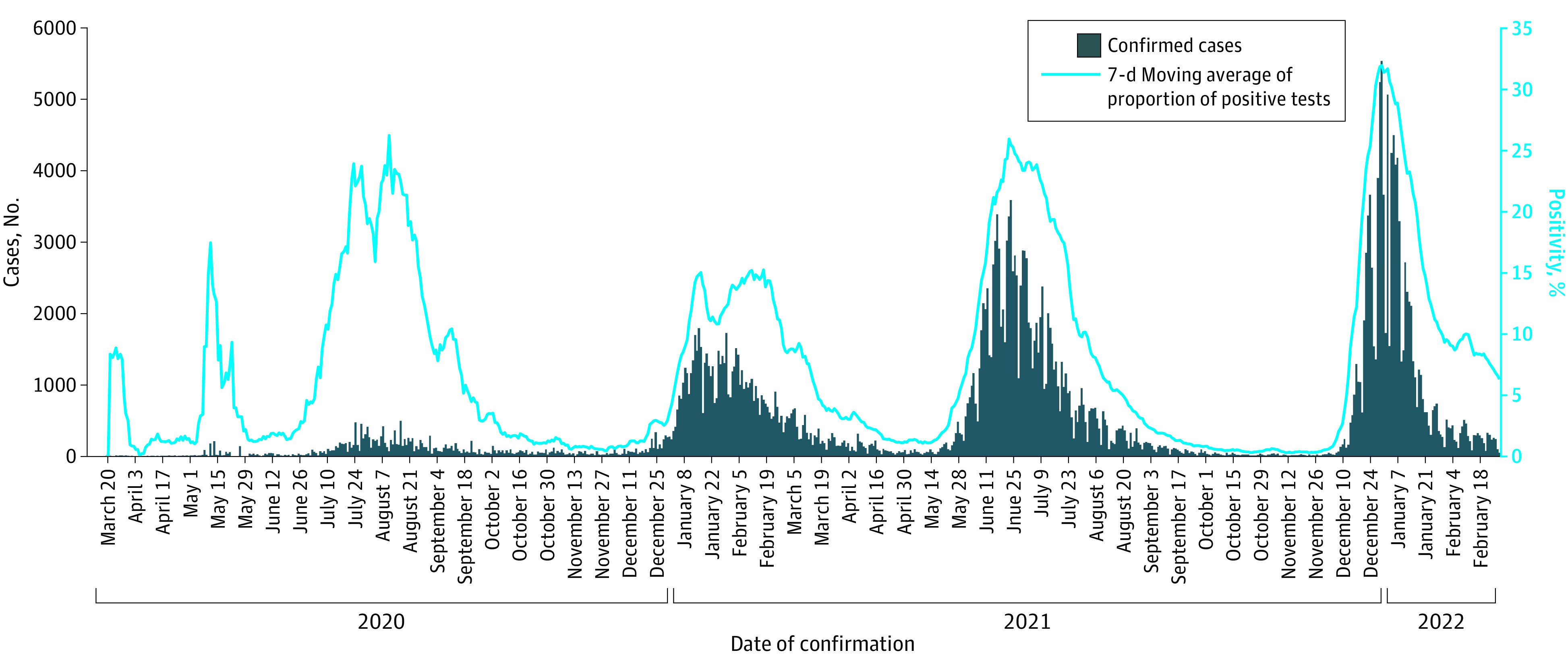
Daily Confirmed SARS-CoV-2 Cases and Percentage of Positivity in Zambia, 2020-2022

National data on daily SARS-CoV-2–positive tests and test positivity rates are collected by the Zambia National Public Health Institute and reported publicly.^22^ Because of fluctuating testing volumes and incomplete data on national hospitalizations, epidemic waves were defined based on visual inspection of an epidemic curve generated using publicly available data (Figure 1).^23^ The start of a wave was defined as the date on which a stable number of daily SARS-CoV-2–positive tests transitioned to an increasing number of positive test results, provided that the increase was followed by a peak and a subsequent decline in daily positive test results. The end of a wave was defined as the date on which a decreasing number of daily SARS-CoV-2–positive test results following a peak transitioned to a stable number of daily positive test results. The increase in cases shown on the epidemic curve between April 29, 2020, and June 8, 2020, was not considered a wave because COVID-19 had not yet spread nationally in Zambia (a high proportion of the SARS-CoV-2 testing conducted during that time was related to an outbreak in the rural Nakonde District). Data on relative variant genome frequency by region from the Global Initiative on Sharing Avian Influenza Data (GISAID) were used to define the SARS-CoV-2 variant that was circulating during each wave.^24^

### COVID-19 Vaccination in Zambia

Zambia began offering COVID-19 vaccination before the start of wave 3 in April 2021. The ChAdOx1-S COVID-19 vaccine (AstraZeneca) was the first vaccine type available in Zambia, followed by Ad26.COV2.S (Janssen), mRNA-127 (Moderna), BNT162b2 (Pfizer-BioNTech), and BBIBP-CorV (Sinopharm). An additional vaccine dose after completing a primary vaccine series (ie, booster) became available in January 2022. Estimates for the proportion of Zambia’s population that were vaccinated against COVID-19 (ie, ≥1 vaccine dose) were less than 1% at the start of wave 3 and 4.5% at the start of wave 4.^25^

### Statistical Analysis

Data analyses were conducted across waves and within waves. Data from patients admitted during nonwave periods were excluded (approximately 9% of study patients). Across-wave analyses focused on descriptive statistics and comparing wave 4, which was dominated by SARS-CoV-2 variant of concern Omicron (B.1.1.529) (a variant widely reported to be less likely to cause severe COVID-19),^6,26,27^ with combined data from waves 1, 2, and 3.

Mixed-effects logistic regression models were used to assess associations between risk factors for poor COVID-19 outcomes and in-hospital mortality in both unadjusted and adjusted models. Risk factors included wave of admission, sex, age, number of self-reported comorbidities, HIV status, COVID-19 severity at admission, and COVID-19 treatment center. Mixed-effects models were used to account for the hierarchical structure of the study data (COVID-19 treatment center included as a random-effects term).

Within-wave analyses were used to investigate whether surges in demand for health services altered the demographic profiles and clinical characteristics of patients admitted to COVID-19 treatment centers. Data from admitted patients were aggregated into 4-week segments within wave periods, with the first segment of each wave beginning on the respective wave’s start date. We assumed, based on anecdotal evidence, that patients admitted during the first 4-week segment of a wave were admitted during a period of baseline health system demand, whereas patients admitted during subsequent 4-week segments were admitted during periods of surging health system demand. Age and COVID-19 disease status for patients admitted during periods of baseline demand (first 4 weeks) were compared with subsequent 4-week segments. Associations between age of 60 years or older and severe COVID-19 at admission and being admitted during a period of baseline demand vs surging demand were assessed using mixed-effects logistic regression models.

Data were analyzed using R software, version 4.02 (R Foundation for Statistical Computing). An α = .05 was used with 2-tailed tests to assess statistical significance.

## Results

### Patients

A total of 312 937 positive SARS-CoV-2 test results were reported in Zambia by the Zambia National Public Health Institute between March 18, 2020, and March 1, 2022. During that same period, 4208 patients were included in the COVID-19 Clinical Outcomes Study, and 3876 (92.1%) were admitted during wave periods (mean [SD] age, 50.6 [19.5] years; 2103 men [54.3%] and 1765 women [45.5%] [data on sex was missing for 8 patients]).

### COVID-19 Waves 

Four waves were identified during the study period (Figure 1). Among patients admitted to COVID-19 treatment centers during those waves and included in the study, wave 3 had the highest number of admissions and the highest number of deaths (Table 1). Compared with waves 1, 2, and 3 (pooled), wave 4 had a significantly lower proportion of patients who were male (416 of 1012 [41.1%] vs 1687 of 2856 [59.1%]), who were 60 years or older (250 of 1009 [24.8%] vs 1116 of 2837 [39.3%]), who had hypertension (249 of 1012 [24.6%] vs 1276 of 2864 [44.6%]), who had severe COVID-19 at admission (346 of 989 [35.0%] vs 2001 of 2807 [71.3%]), and who died (131 of 909 [14.4%] vs 700 of 2682 [26.1%]). The proportion of HIV-infected patients did not differ between wave 4 (167 of 920 [18.2%]) and waves 1, 2, and 3 (421 of 2300 [18.3%]).

**Table 1.** Demographic and Clinical Variables by Wave for Patients Admitted to Participating COVID-19 Treatment Centers in Zambia, 2020-2022^a^^,^^b^

Variable	Wave 1 (ancestral lineages; June 27 to October 5, 2020) (n = 558)	Wave 2 (Beta variant [B.1.351]; December 22, 2020, to May 10, 2021) (n = 1052)	Wave 3 (Delta variant [B.1.617]; May 24 to September 1, 2021) (n = 1254)	Wave 4 (Omicron variant [B.1.1.529]) October 12, 2021, to February 5, 2022) (n = 1012)	*P* value^c^
Sex					
Male	349/558 (62.5)	625/1046 (59.8)	713/1252 (56.9)	416/1012 (41.1)	<.001
Female	209/558 (37.5)	421/1046 (40.2)	539/1252 (43.1)	596/1012 (58.9)	
Age, mean (SD), y	48.9 (16.3)	54.0 (16.4)	55.5 (18.0)	42.1 (22.5)	<.001
Age group, y					
<30	72/558 (12.9)	76/1030 (7.4)	110/1249 (8.8)	364/1009 (36.1)	<.001
30-39	87/558 (15.6)	116/1030 (11.3)	155/1249 (12.4)	211/1009 (20.9)	
40-49	143/558 (25.6)	198/1030 (19.2)	197/1249 (15.8)	103/1009 (10.2)	
50-59	106/558 (19.0)	231/1030 (22.4)	230/1249 (18.4)	81/1009 (8.0)	
≥60	150/558 (26.9)	409/1030 (39.7)	557/1249 (44.6)	250/1009 (24.8)	
HIV infection	106/447 (23.7)	153/913 (16.8)	162/940 (17.2)	167/920 (18.2)	.96
Active tuberculosis	18/558 (3.2)	17/1052 (1.6)	22/1254 (1.8)	38/1012 (3.8)	.008
Diabetes	95/558 (17.0)	226/1052 (21.5)	257/1254 (20.5)	72/1012 (7.1)	<.001
Hypertension	203/558 (36.4)	511/1052 (48.6)	562/1254 (44.8)	249/1012 (24.6)	<.001
No. of self-reported comorbidities^d^					
0	208/558 (37.3)	342/1052 (32.5)	460/1254 (36.7)	475/1012 (46.9)	<.001
1-2	261/558 (46.8)	600/1052 (57.0)	667/1254 (53.2)	446/1012 (44.1)	
≥3	89/558 (15.9)	110/1052 (10.5)	127/1254 (10.1)	91/1012 (8.99)	
Severe disease at admission^e^	303/557 (54.4)	802/1045 (76.7)	896/1205 (74.4)	346/989 (35.0)	<.001
Deaths^f^	91/549 (16.6)	217/980 (22.1)	392/1153 (34.0)	131/909 (14.4)	<.001
Vaccinated^g^	NA	NA	45/1254 (3.6)	182/1012 (17.9)	<.001

Abbreviation: NA, not applicable.

^a^ Data are presented as number and total number (percentage) of patients unless otherwise indicated. Patients admitted during nonwave periods were not included: before wave 1: 136; after wave 1: 79; after wave 2: 54; after wave 3: 50; and after wave 4: 13.

^b^ In cases where the denominator is less than the wave-specific sample size, data were missing.

^c^ Comparison of wave 4 and waves 1, 2, and 3 (pooled). The *P* value for age group refers to 60 years or older vs younger than 60 years. The *P* value for number of self-reported comorbidities refers to 0 comorbidities vs 1 or more comorbidities. The χ^2^ test was used for proportions and the Wilcoxon rank sum test for means. Statistical tests do not account for site-level clustering.

^d^ Comorbidities were self-reported and included cardiac disease, hypertension, diabetes, other pulmonary disease, active tuberculosis, previous tuberculosis, asthma, kidney disease, liver disease, neurologic disorder, asplenia, malignant neoplasm, and current smoking.

^e^ Severe disease at admission is a composite variable for disease status at admission. Severe COVID-19 was defined as 1 or more of the following conditions: oxygen saturation below 90%, respiratory rate greater than 30 breaths/min, and need for oxygen therapy. All other patients were deemed to have mild to moderate COVID-19.

^f^ Deaths indicate the mortality status of patients in the hospital (does not include deaths outside the hospital).

^g^ Vaccinated indicates 1 or more COVID-19 vaccine doses at admission. Vaccines available in Zambia during this period were Ad26.COV2.S (Janssen), mRNA-127 (Moderna), BNT162b2 (Pfizer-BioNTech), and BBIBP-CorV (Sinopharm).

### Risk Factors for In-Hospital Mortality

In the fully adjusted model, admission during wave 2 had significantly lower odds of in-hospital death (compared with wave 4) (adjusted odds ratio [aOR], 0.58; 95% CI, 0.42-0.80), and admission during wave 3 had higher odds of in-hospital death (compared with wave 4) (aOR, 1.15; 95% CI, 0.85-1.55), although the latter association was not significant (Table 2). The fully adjusted model also indicated that patients across all waves who were male (aOR, 1.64; 95% CI, 1.34-2.02), were 60 years or older (aOR, 3.55; 95% CI, 2.34-5.52), had comorbidities (1-2 comorbidities: aOR, 1.52; 95% CI, 1.19-1.94; ≥3 comorbidities: aOR, 2.27; 95% CI, 1.65-3.14), had severe COVID-19 at admission (aOR, 7.20; 95% CI, 5.24-10.07), or had HIV infection (aOR, 1.39; 95% CI, 1.07-1.79) had significantly higher odds of in-hospital mortality compared with their respective reference categories.

**Table 2.** Risk Factors and Association With In-Hospital Death

Risk factor	aOR (95% CI)	
	Model adjusted for treatment center only	Fully adjusted model^a^
Admission by COVID-19 wave		
Wave 1	0.96 (0.70-1.31)	0.69 (0.47-1.00)
Wave 2	1.60 (1.25-2.04)	0.58 (0.42-0.80)
Wave 3	3.20 (2.55-4.01)	1.15 (0.85-1.55)
Wave 4	1 [Reference]	1 [Reference]
Sex		
Male	1.75 (1.50-2.06)	1.64 (1.34-2.02)
Female	1 [Reference]	1 [Reference]
Age group, y		
<30	1 [Reference]	1 [Reference]
30-39	1.72 (1.13-2.66)	1.19 (0.72-2.00)
40-49	3.12 (2.13-4.65)	1.28 (0.79-2.10)
50-59	4.76 (3.3-7.01)	1.81 (1.14-2.93)
≥60	9.67 (6.93-13.86)	3.55 (2.34-5.52)
HIV		
Infection	1.11 (0.90-1.38)	1.39 (1.07-1.79)
No infection	1 [Reference]	1 [Reference]
No. of self-reported comorbidities		
0	1 [Reference]	1 [Reference]
1-2	2.32 (1.94-2.78)	1.52 (1.19-1.94)
≥3	3.65 (2.84-4.70)	2.27 (1.65-3.14)
COVID-19 at admission		
Severe	12.15 (9.40-15.97)	7.20 (5.24-10.07)
Mild	1 [Reference]	1 [Reference]

Abbreviation: aOR, adjusted odds ratio.

^a^ Fully adjusted model includes wave (1-4), sex (male or female), age (<30, 30-39, 40-49, 50-59, or ≥60 years), comorbidity burden (0, 1-2, or ≥3), disease status at admission (mild or severe), COVID-19 vaccination status (vaccinated or not vaccinated), and COVID-19 treatment center (random-effects term).

### COVID-19 Treatment Center Admissions Dynamics During Waves

**Figure 2.**
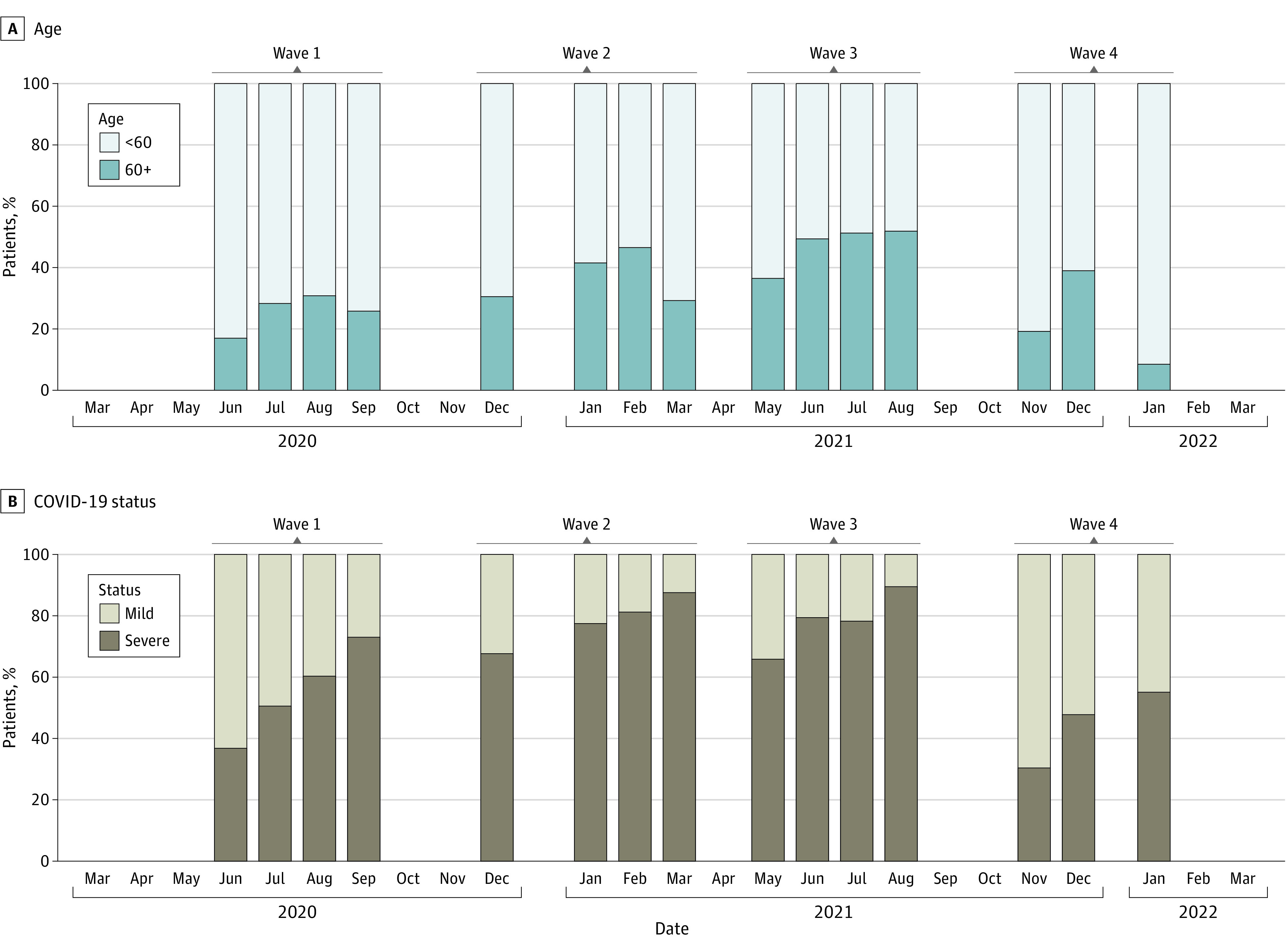
Proportion of Patients Admitted During 4-Week Segments Within Waves by Age and COVID-19 Status at Admission

Inspection of trends across all 4 waves indicated that increased proportions of patients at high risk for poor COVID-19 outcomes (ie, ≥60 years of age and/or with severe COVID-19 at admission) were admitted during segments that fell in the middle or, in some cases, at the end of waves vs at the beginning (Figure 2). Indeed, regression modeling that used combined data indicated that patients admitted during weeks 5 to 9 and weeks 10 to 13 had significantly higher odds of being 60 years or older (aOR, 2.09 [95% CI, 1.79-2.45]) at weeks 5-9 and 2.12 [95% CI, 1.72-2.61] at weeks 10-13) or having severe COVID-19 at admission (aOR, 2.49 [95% CI, 2.14-2.90] at weeks 5-9 and 2.90 [95% CI, 2.34-3.61] at weeks 10-13) than patients admitted during the first 4 weeks of a wave (Table 3). This was also true in the wave-specific models for waves 1, 2, and 3 and for weeks 5 through 9 (vs weeks 1 through 4) during wave 4 (Table 3).

**Table 3.** Demographic and Clinical Characteristics and Association With Admission Timing During Waves

Timing of patient admission	Age ≥60 y		Severe COVID-19 at admission	
	No. (%) of patients	aOR (95% CI)	No. (%) of patients	aOR (95% CI)
Combined model (includes patients from all 4 waves)^a^				
Weeks 1-4	380/1459 (26.0)	1 [Reference]	669/1428 (46.8)	1 [Reference]
Weeks 5-9	694/1646 (42.2)	2.09 (1.79-2.45)	1128/1632 (69.2)	2.49 (2.14-2.90)
Weeks 10-13	239/573 (41.7)	2.12 (1.72-2.61)	413/567 (72.8)	2.90 (2.34-3.61)
Weeks 14-17	53/168 (31.5)	1.36 (0.95-1.92)	137/168 (81.5)	5.04 (3.38-7.76)
**Wave-specific models^a^**					
Wave 1				
Weeks 1-4	15/89 (16.9)	1 [Reference]	32/88 (36.4)	1 [Reference]
Weeks 5-9	60/213 (28.2)	1.88 (1.02-3.64)	108/213 (50.7)	1.92 (1.15-3.25)
Weeks 10-13	56/182 (30.8)	2.13 (1.15-4.16)	109/182 (59.9)	2.78 (1.65-4.80)
Weeks 14-17	19/74 (25.7)	1.66 (0.78-3.60)	54/74 (73.0)	5.04 (2.59-10.1)
Wave 2				
Weeks 1-4	60/198 (30.3)	1 [Reference]	141/209 (67.5)	1 [Reference]
Weeks 5-9	217/523 (41.5)	1.63 (1.15-2.35)	406/526 (77.2)	1.64 (1.11-2.42)
Weeks 10-13	113/244 (46.3)	2.01 (1.36-3.02)	197/244 (80.7)	2.03 (1.28-3.26)
Weeks 14-17	19/65 (29.2)	1.02 (0.54-1.89)	58/66 (87.9)	3.49 (1.55-8.97)
Wave 3				
Weeks 1-4	172/473 (36.4)	1 [Reference]	293/446 (65.7)	1 [Reference]
Weeks 5-9	301/612 (49.2)	1.55 (1.20-1.99)	477/601 (79.4)	1.89 (1.41-2.54)
Weeks 10-13	69/135 (51.1)	1.73 (1.16-2.58)	101/130 (77.7)	1.74 (1.08-2.87)
Weeks 14-17	15/29 (51.7)	1.58 (0.73-3.43)	25/28 (89.3)	2.96 (0.97-12.91)
Wave 4				
Weeks 1-4	133/699 (19.0)	1 [Reference]	203/687 (29.5)	1 [Reference]
Weeks 5-9	116/298 (38.9)	2.71 (2.00-3.76)	137/291 (47.1)	2.28 (1.70-3.07)
Weeks 10-13	1/12 (8.3)	0.39 (0.02-2.09)	6/11 (54.5)	3.99 (1.11-15.14)

Abbreviation: aOR, adjusted odds ratio.

^a^ Model adjusted for COVID-19 treatment center (random-effects term).

## Discussion

Demographic profiles, clinical characteristics, and outcomes of patients admitted to COVID-19 treatment centers varied across 4 distinct COVID-19 waves in Zambia. Within waves, we observed a consistent pattern in which the proportion of patients at high risk for poor COVID-19 outcomes increased as waves progressed. This finding was likely indicative of admission prioritization necessitated by a combination of surges in demand for health services and Zambia’s limited health system capacity. These findings are among the first to explore this aspect of the COVID-19 epidemic and support the urgent need to expand health system capacity and improve health system resiliency in Zambia and in other countries with resource-limited health systems.

As reported elsewhere,^6,15,26,27^ patients admitted to COVID-19 treatment centers during wave 4, when the SARS-CoV-2 Omicron variant was circulating in Zambia, were younger, less likely to have comorbidities, and less likely to have severe COVID-19 at admission. These findings appear to be consistent with observational data that show reduced risk of severe disease with Omicron infection,^15,26,27^ although the inclusion of patients with incidental COVID-19 infections likely also affected the findings.^26^ It remains unclear whether the reduced risk of severe disease with Omicron infection reflects partial immunity from vaccination and/or prior infection or whether it is associated with reduced pathogenicity of the Omicron variant.^28,29^

Beyond wave of admission, other risk factors associated with in-hospital death at COVID-19 treatment centers in Zambia were consistent with previously reported data.^30,31^ Our finding that HIV infection was significantly associated with in-hospital death is particularly relevant for Zambia, which has an HIV prevalence of 11.1% among persons aged 15 to 49 years,^32^ as well as other countries in the sub-Saharan region of Africa, which has the world’s highest burden of persons living with HIV.^33^ Factors that drive this association could be related to poorly controlled HIV infection,^16^ resulting in immunosuppression and opportunistic infections, as well as residual confounding related to higher risk for noncommunicable diseases in persons living with HIV.^34,35^

Within-wave data indicating that the proportion of high-risk patient admissions increased during waves suggest that there was prioritization of admissions during periods of surging demand for health services. The apparent prioritization, which was likely necessitated by limited capacity at Zambia’s COVID-19 treatment centers, likely had a direct effect on lower-risk patients seeking COVID-19 treatment center admission. Those lower-risk patients may have been admitted during periods of baseline demand but, because of prioritization, were likely not admitted during periods of surging demand. Although the health outcomes of these patients are unknown, without the care provided in COVID-19 treatment centers, it is possible that they experienced high levels of morbidity and even mortality. Indeed, reports noted that increases in community deaths and all-cause mortality aligned with COVID-19 waves in Zambia.^36,37^

Addressing health system capacity limitations and implementing practices to improve health system resiliency are critical as Zambia prepares for upcoming COVID-19 waves and future epidemics. Expansion of health system infrastructure is needed, including additional health facilities, hospital beds, ventilators, and oxygen production facilities. Added investments in human resources are similarly needed for the country to develop a larger and better-trained workforce capable of responding to the needs presented by epidemics such as COVID-19. Use of virtual platforms to conduct trainings for health workers is one approach that is already in use in Zambia^38^ but could benefit from additional resources and wider adoption. Additional practices can also be implemented to improve health system resiliency as it relates to COVID-19. These practices include continuing to implement infection prevention and control practices, using home-based care in lieu of unnecessary hospitalizations, establishing and expanding access for targeted antivirals, and continuing to expand SARS-CoV-2 vaccination. Although the study described in this article was conducted in Zambia, it is likely that resource-limited health systems in other countries experienced challenges when faced with surging demand for health services during COVID-19 waves. Understanding those challenges and the country-specific measures needed to address them will be critical as countries work to develop higher-capacity and more resilient health systems.

### Limitations

The findings of this study are subject to at least 6 limitations. First, we used GISAID to correlate SARS-CoV-2 variants with specific waves rather than sequencing specimens from patients in the study. Second, reliable data were not available on national COVID-19–related hospitalization numbers or COVID-19 hospitalizations by facility, which limits our understanding of the reach and generalizability of this study and prevents us from reporting changes in hospitalization rates across waves. Third, this study was performed in hospitalized patients and thus subject to collider bias,^39^ which might limit the generalizability of these findings beyond hospitalized patients. Fourth, even though we controlled for available covariates, confounding may still be present in our model that assessed risk factors for in-hospital mortality. Fifth, it is possible that the findings from our within-wave analyses were influenced by the use of 4-week segments or reflect the timing of COVID-19 transmission dynamics (eg, spread of infection among specific groups at specific time points during waves) rather than prioritization of treatment center admission toward high-risk patients. Sixth, as with many studies conducted during emergency responses, data completeness was a limitation because staff who were responsible for data collection were also responding to other urgent demands at the COVID-19 treatment centers.

## Conclusions

Although the changes in epidemiologic data across waves described in this cohort study largely reflect those reported elsewhere, within-wave analyses revealed a pattern in which surges in demand for health services during COVID-19 waves in Zambia aligned with a prioritization of treatment center admissions toward higher-risk patients. This pattern, which was likely driven by the limited capacity of Zambia’s health system, underscores the need to expand health system capacity and strengthen health system resiliency in Zambia and other countries to prepare for future epidemics.

## Notes

Supplement.
Data Sharing Statement
