# Supplementary material for: Clinical Characteristics and Outcomes of Patients Hospitalized With COVID-19 During the First 4 Waves in Zambia
Source: JAMA Netw Open. 2022 Dec 13;5(12):e2246152. doi: 10.1001/jamanetworkopen.2022.46152 (PMC9856263; doi:10.1001/jamanetworkopen.2022.46152)
Supplement: Supplement. — Data Sharing Statement [file jamanetwopen-e2246152-s001.pdf]

Data Sharing Statement

Minchella. Clinical Characteristics and Outcomes of Patients Hospitalized With COVID-19 During the First 4 Waves in Zambia. *JAMA Netw Open*. Published December 13, 2022. doi:10.1001/jamanetworkopen.2022.46152

Data
Data available: No

Additional Information
Explanation for why data not available: Data are owned by the Zambian Ministry of Health. Requests for data access can be made to Zambia MoH.
